# Functional annotations of diabetes nephropathy susceptibility loci through analysis of genome-wide renal gene expression in rat models of diabetes mellitus

**DOI:** 10.1186/1755-8794-2-41

**Published:** 2009-07-09

**Authors:** Yaomin Hu, Pamela J Kaisaki, Karène Argoud, Steven P Wilder, Karin J Wallace, Peng Y Woon, Christine Blancher, Lise Tarnow, Per-Henrik Groop, Samy Hadjadj, Michel Marre, Hans-Henrik Parving, Martin Farrall, Roger D Cox, Mark Lathrop, Nathalie Vionnet, Marie-Thérèse Bihoreau, Dominique Gauguier

**Affiliations:** 1 The Wellcome Trust Centre for Human Genetics, University of Oxford, Roosevelt Drive, Oxford OX3 7BN, UK; 2 Steno Diabetes Center, Copenhagen, Denmark; 3 Department of Medicine, Division of Nephrology, Helsinki University Central Hospital and Folkhälsan Institute of Genetics, Helsinki, Finland; 4 CHU Poitiers, University Hospital, Endocrinology and INSERM, ERM 324, Poitiers, France; 5 Department of Diabetology, Bichat Hospital and INSERM, U695, Xavier Bichat University of Medicine, Paris, France; 6 Department of Medical Endocrinology, Rigshospitalet, University Hospital of Copenhagen, Copenhagen, Denmark; 7 MRC Mammalian Genome Unit, Harwell OX11 0RD, UK; 8 National Genotyping Centre, Evry, France; 9 INSERM, UMR S 525, Université Pierre et Marie Curie-Paris 6, Paris, France; 10 INSERM, U872, Centre de Recherche des Cordeliers, Paris, France

## Abstract

**Background:**

Hyperglycaemia in diabetes mellitus (DM) alters gene expression regulation in various organs and contributes to long term vascular and renal complications. We aimed to generate novel renal genome-wide gene transcription data in rat models of diabetes in order to test the responsiveness to hyperglycaemia and renal structural changes of positional candidate genes at selected diabetic nephropathy (DN) susceptibility loci.

**Methods:**

Both Affymetrix and Illumina technologies were used to identify significant quantitative changes in the abundance of over 15,000 transcripts in kidney of models of spontaneous (genetically determined) mild hyperglycaemia and insulin resistance (Goto-Kakizaki-GK) and experimentally induced severe hyperglycaemia (Wistar-Kyoto-WKY rats injected with streptozotocin [STZ]).

**Results:**

Different patterns of transcription regulation in the two rat models of diabetes likely underlie the roles of genetic variants and hyperglycaemia severity. The impact of prolonged hyperglycaemia on gene expression changes was more profound in STZ-WKY rats than in GK rats and involved largely different sets of genes. These included genes already tested in genetic studies of DN and a large number of protein coding sequences of unknown function which can be considered as functional and, when they map to DN loci, positional candidates for DN. Further expression analysis of rat orthologs of human DN positional candidate genes provided functional annotations of known and novel genes that are responsive to hyperglycaemia and may contribute to renal functional and/or structural alterations.

**Conclusion:**

Combining transcriptomics in animal models and comparative genomics provides important information to improve functional annotations of disease susceptibility loci in humans and experimental support for testing candidate genes in human genetics.

## Background

Diabetes mellitus (DM) is a growing cause of end stage renal disease in developed countries. Diabetic nephropathy (DN) develops in 35–40% of diabetic patients as the result of intrarenal metabolic, hemodynamic and structural changes [1,2]. DN is a complex phenotype caused by the combined effects of susceptibility alleles and environmental factors which contribute to poor glycaemic control and hypertension [3]. The importance of genetic factors in DN is suggested by epidemiological studies and by the familial clustering of nephropathy in both type 1 and type 2 DM [4-6]. Nephropathy does not necessarily develop in a significant proportion of diabetic patients, suggesting the involvement of specific genes.

Genetic studies of nephropathy in diabetic patients are hampered by heterogeneous clinical features, including frequent association with hypertension, and between-study variation in design and phenotype assessment [7]. Although genome-wide linkage studies have identified regions of the human genome contributing to DN [8-12], the causative genes remain unknown. Association studies have been carried out with DN candidate genes that regulate blood pressure, the synthesis and degradation of renal structural components, the metabolism and transport of glucose and the process of advanced glycation [1,13,14]. High-throughput transcription profiling technologies, which are powerful tools for determining gene expression regulation in health and disease situations, can enrich genome annotations and uncover new DN candidates [14-16]. Such studies in patients and controls pose technical, scientific and ethical issues [17].

Animal models of DM are essential components of genetic and functional genomic investigations. They include animals made insulin-deficient by injection with the toxin streptozotocin (STZ) and inbred models of spontaneous DM [18]. The inbred Goto Kakizaki (GK) rat shows genetically determined alterations of glucose tolerance and insulin secretion [19], and renal structural changes similar to those observed in the early phase of DN, including thickening of the glomerular and tubular basement membranes, and glomerular hypertrophy [20,21]. Elevation of glomerular macrophage infiltration is present whereas proteinuria has not been consistently observed. In contrast STZ-treated animals show little evidence of renal histopathology limited to mild glomerular mesangial expansion, which is correlated with blood glucose levels and proteinuria [22,23]. The genetic basis of these renal phenotypes in these diabetic models has not been investigated. These models underlie clearly different etiological and pathological contexts of DM, and provide complementary tools to document hyperglycaemia-induced gene expression changes that can contribute to the development of renal structural and functional anomalies relevant to DN.

In this study, we have investigated the effects of prolonged hyperglycaemia on genome-wide renal gene expression regulation in GK rats and inbred rats of similar genetic origin (Wistar-Kyoto, WKY) made severely diabetic by STZ, which may be caused by differential adaptations to mild or severe hyperglycaemia induced by genetic variants in GK and environmental changes in both diabetic models. We specifically demonstrated the possibility to use data from both comparative genomics and gene transcription regulation to test transcriptional responsiveness of genes to hyperglycaemia and renal structural changes that can improve functional annotations of known and potentially novel positional candidate genes localised in DN susceptibility loci.

## Methods

### Animals

Rats of the GK strain from the Oxford colony (GK/Ox) were used. WKY control rats were purchased from a commercial supplier (Harlan, UK). All animal procedures complied with the University of Oxford's Local Ethical Review Process and were carried out under project licences 30/2001 and 30/2324 granted by the UK Home Office. A group of 3 month old WKY rats was injected intravenously with a solution of Streptozotocin (STZ) (Sigma-Aldrich, Poole, UK) at 75 mg/kg in citrate buffer (pH 4.5) to induce permanent and severe insulin deficiency and hyperglycaemia of about 16.6 mM. This group is referred as STZ-WKY. Experiments were performed with male rats fed standard laboratory chow pellets (B&K Universal, Hull, UK) and kept on 12 h light/dark cycle. Body weight and plasma glucose were monitored. GK and WKY rats were killed at 3 months by CO_2 _asphyxiation, whereas STZ-WKY rats were killed at 6 months to ensure that duration of hyperglycaemia in both diabetic models was similar (ie. 3 months). WKY rats were used as pre STZ-treated controls for the STZ-WKY group. The left kidney was removed, snap-frozen in liquid nitrogen and stored at -80°C until RNA preparation. The right kidney was fixed in Dubosq-Brazil (0.4% picric acid, 27% formalin, 7% acetic acid, 54% ethanol) and embedded in paraffin. Sections (3 μm) were stained with periodic acid-Schiff (PAS) reagent and counterstained with hematoxylin (Sigma-Aldrich, Poole, UK). Presence of renal anomalies in diabetic rats was evaluated by examining 3 sections per rat.

### RNA preparation

Total kidney RNA samples were individually prepared from four (GK, WKY) or five (STZ-WKY) animals per group. Total RNA was extracted twice using Trizol reagents (Invitrogen Life Technologies, Paisley, UK) and cleaned with RNeasy columns (Qiagen Ltd., Crawley, UK). RNA quality was determined with an Agilent 2100 Bioanalyzer (Agilent Technologies, Waldbronn, Germany).

### Hybridization to Affymetrix gene expression arrays

Synthesis of cDNA and biotin-labeled cRNA (Affymetrix ltd., High Wycombe, UK) was performed using 14 μg of RNA. Biotinylated cRNA (15 μg) were fragmented and individually hybridized to GeneChip Rat expression Arrays 230A containing 15,866 probe sets (Affymetrix ltd., High Wycombe, UK). Best quality samples from three animals per group were used and individually hybridized to the arrays. Washing and staining procedures were performed using a Fluidics Station 450 according to manufacturer's protocol (Affymetrix ltd, High Wycombe, UK). Arrays were scanned at 560 nm using an array scanner (Agilent Technologies, Waldbronn, Germany).

### Hybridization to Illumina gene expression arrays

Replication experiments were carried out with Illumina arrays, a bead-based technology that is different from that of Affymetrix. Double-stranded cDNA and biotin-labelled cRNA were synthesized from RNA (300 ng) using the Illumina^® ^TotalPrep RNA amplification it (Ambion Inc., Austin, TX). Each biotinylated cRNA (750 ng) was hybridized to prototype Sentrix^® ^BeadChip RatRef-12_V1_Eval Whole-Genome Gene Expression arrays containing 22,636 oligonucleotides (Illumina Inc., San Diego, CA). For each rat group, individual hybridizations were performed in technical duplicates with 4 biological replicates, including the three samples previously used for the Affymetrix experiments. Following washing steps and staining with Streptavidin-Cy3, arrays were scanned on the Illumina^® ^BeadArray Reader and the images analyzed using the Illumina BeadStudio software.

Experiments are MIAME compliant. Protocols and data are available through ArrayExpress  under the accession E-MEXP-1195 (Affymetrix) and E-TABM-502 (Illumina).

### Quantitative real time PCR

First-strand cDNA were synthesized from total RNA with Superscript II cDNA synthesis kits (Invitrogen Gibco, Paisley, UK). QRT-PCR was performed with oligonucleotides designed to span an intron/exon boundary (see Additional file 1) using a Rotor-Gene 3000™ system (Corbett Research, Milton, UK) and QuantiTect SYBR Green PCR kits (Qiagen Ltd, Crawley, UK). Experiments were performed with biological quadruplicates (ie. the four samples used for the Illumina experiments) and technical triplicates. Analyses were performed using the Rotor-Gene software (Corbett Research, Milton, UK). Gene dosage was calculated with the standard curve generated and normalized to actin.

### Gene transcription data analysis

The renal Affymetrix and Illumina gene expression datasets were previously used alongwith other rat and mouse datasets to test and optimise appropriate methods for analysis, including data extraction and normalisation procedures [24]. Analysis of Affymetrix CEL file data was performed using the Bioconductor packages in the R language and environment as previously described [25]. Briefly, Affymetrix data were normalized using the RMA method and the linear model for microarray data (LIMMA) package was used to assess significance between groups. For Illumina, microarray results were normalized by quantile normalization and LIMMA was used to calculate significant differences between groups using slide number as a covariate to correct for chip-specific effects. Affymetrix and Illumina datasets were used independently to carry out statistical data analyses of gene expression changes between rat groups. Cross-platform consistency of statistical significance and fold change of renal gene expression have been reported elsewhere [24]. For both Affymetrix and Illumina datasets, P-values were corrected for multiple testing using a 5% false discovery rate as the cut-off for significance. Data from QRT-PCR-based gene transcription analysis were analysed using SPSS version 16.0 statistical package. A Bonferroni post hoc test was used to assess differences between the diabetic models and the normoglycemic WKY strain as well as between the two diabetic models. A *p *value of less than 0.05 was considered to be statistically significant for pairwise comparisons.

## Results

### Pathophysiological features in GK and STZ-WKY rats

The effect of prolonged diabetes on gene expression changes was determined in situations of similar durations (3 months) of mild spontaneous (GK) or severe experimentally-induced (STZ-WKY) hyperglycaemia. Glycaemia in STZ-WKY rats was monitored to be greater than 13.5 mM and not to exceed 16.6 mM throughout of the 3 months of the experiment. Glycaemia was significantly more elevated in GK and STZ-WKY rats than in WKY controls (Additional file 2). Body weight was similar in GK and WKY rats. Prolonged severe hyperglycaemia in 6 months old STZ-WKY rats resulted in marked decreased body weight when compared to 3 months old WKY controls

The two diabetic models showed mild renal histopathological changes, which may be relevant to early pathological events in DN progression. When compared to age-matched WKY (Additional file 3A), GK rats showed glomerular alterations including basement membrane thickening, matrix accumulation of hyaline, indicating mesangial extracellular matrix expansion, and increased capillary luminal volume suggesting glomerular hypertrophy (Additional files 3B3 and 3C). Tubular basement membrane thickening was observed in both collecting and distal tubules of GK sections. Following three months of severe hyperglycaemia, STZ-WKY rats exhibited glomerular hypertrophy associated with capillary loop expansion and to a lesser extend basement membrane thickening and mild mesangial expansion (Additional files 3D–F).

### Affymetrix-based genome-wide renal transcription profiles in diabetic rats

#### Overview of gene transcription changes

**Figure 1:**
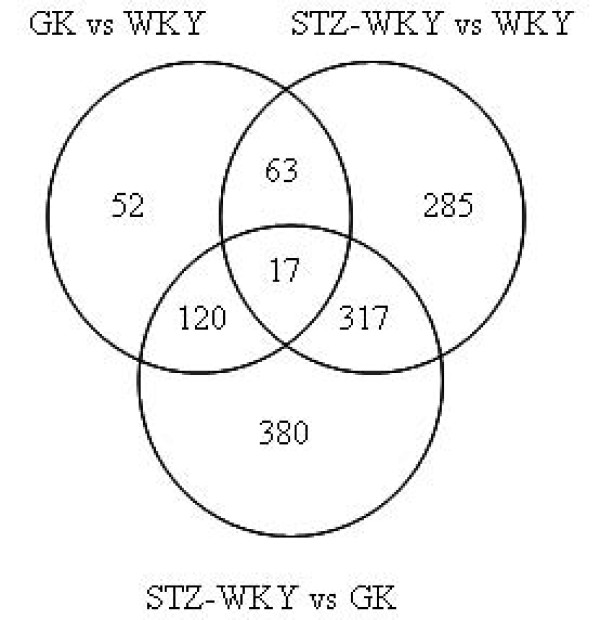
**Differentially expressed genes in kidneys of GK and STZ-induced diabetic rat models**. Number of Affymetrix probesets corresponding to genes statistically differentially expressed between models (p < 0.05) are shown. The full list of differentially expressed genes between the three models is given in Additional file 4.

**Figure 2:**
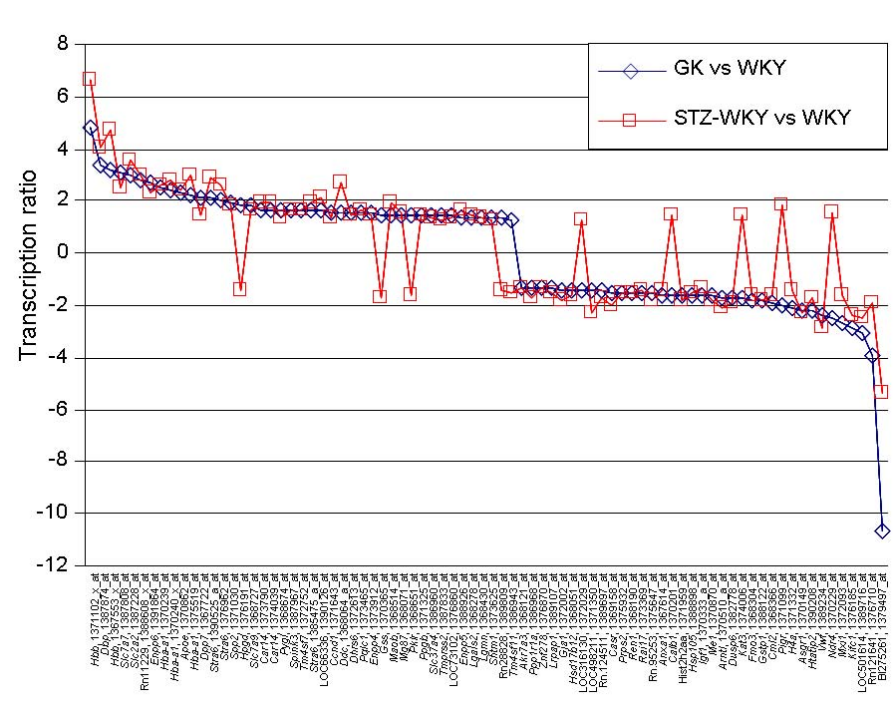
**Patterns of transcription for genes consistently differentially expressed between diabetic rats (GK and STZ-WKY) and WKY controls**. Genes are listed along the X axis with both Affymetrix probe set names and either symbols for known genes or accession number references for EST sequences. Genes are ranked from the highest (left) to the lowest (right) changes in transcription ratio in the GK vs WKY comparison.

Pairwise comparisons of microarray data between rat groups sharing similar genetic backgrounds of Wistar origin were performed to provide detailed information on transcriptional responses to prolonged and permanent hyperglycaemia (GK or STZ-WKY compared to WKY), as well as specific changes caused by gene variants predisposing to diabetes and possibly kidney histopathology (GK compared to WKY) and underlying hyperglycaemia severity (GK compared to STZ-WKY). Of the probe sets representing known and predicted transcripts, 252 were found differentially regulated between GK and WKY rats and over 650 between STZ-WKY and WKY, indicating a greater impact of severe hyperglycaemia on renal transcriptional changes (Figure 1). Over 800 genes were differentially expressed between STZ-WKY and GK suggesting that renal transcriptome adaptations to hyperglycaemia in these models involve different mechanisms. Furthermore, when compared to WKY, diabetic models shared transcriptional changes for only 74 genes (corresponding to 80 probesets), which generally showed the same pattern of expression regulation (Figure 2 and additional file 4). In all comparisons there were similar numbers of genes up- and down-regulated. There was no evidence of genomic clustering of differentially expressed genes (data not shown). Pathway analysis identified gene expression mechanisms either consistently affected in both diabetic models or predominantly altered in the GK or STZ-WKY rats (Additional file 5).

#### Transcription regulation of functional candidate genes in diabetic models

To gain insight into DN candidates, expression regulation of individual genes was analyzed. Evidence of significant differential expression in at least one of the comparisons between the three models was found for 1,233 Affymetrix probe sets representing 713 known genes and 468 expressed sequence tags (ESTs) and predicted genes (see Additional file 4). Owing to their relevance to mechanisms involved in DN [26], a number of these genes have already been tested for evidence of genetic association to DN [13,27,28], including genes encoding the angiotensin converting enzyme (*Ace*), angiotensinogen (*Agt*), angiogenin ribonuclease A family 5 (*Ang*), apolipoprotein E (*Apoe*), bone morphogenetic protein 7 (*Bmp7*), carnosinase 1 (*Cndp1*), collagens type IV (*Col4a1*, *Col4a4*), connective tissue growth factor (*Ctgf*), cathepsin L (*Ctsl*), epidermal growth factor (*Egf*), gremlin 1 (*Grem1*), integrin, alpha 1 (*Itga1*), laminin beta 1 (*Lamb1*), lectin galactoside-binding soluble 3 (*Lgals3*), lipoprotein lipase (*Lpl*), matrix metalloproteinase 9 (*Mmp9*), nidogen (Nid), nitric oxide synthase (*Nos*), neuropilin 1 (*Nrp1*), renin (*Ren*), SA hypertension-associated homolog (*Sah*), glucose transporter 2 (*Slc2a*2), a sodium/chloride transporter (*Slc12a3*), hepatic transcription factor 2 (*Tcf2*, *Hnf1b1*), transcobalamin 2 (*Tcn2*), transforming growth factor beta 1-induced transcript 4 (*Tgfb1i4*) and transmembrane 4 superfamily member 4 (*Tm4sf4*). Related genes and other candidates previously tested in DN genetics [13] were found differentially expressed between models, including genes encoding cathepsins (*Ctsb*, *Ctsf*), carboxypeptidases (*Cpe*, *Cpn1*), methylenetetrahydrofolate dehydrogenase (*Mthfd1*) and syndecan 1 (*Sdc1*), which were consistently upregulated in STZ-WKY when compared to WKY, and genes for carnosinase 2 (*Cndp2*), ectonucleotide phosphodiesterase 5 (*Enpp5*), laminin alpha (*Lama1*), beta-galactoside binding lectins (*Lgals1*, *Lgals2*, *Lgals5*, *Lgals3bp*), matrix metalloproteinases (*Mmp11*, *Mmp14*) and a matrix-associated regulator of chromatin (*Smarca4*).

Altered transcription regulation between models was also found for genes involved in extracellular matrix turnover (*Cdh1*, *Cdh16*, *Cdh17, Celsr2, Cyr61, Dag1, Mucdhl, Omd, Sparc, Spon1*) and biochemical processes including the polyol pathway (*Akr1b4, Akr1b8, Akr1c12, Akr7a3, Sord*) and gluthatione metabolim (*Gss*, *Gstm1*, *Gsto1*, *Gstp1*, *Gstt2*, *Hagh*, *Yc2*) (Additional file 4) which are relevant to DN. However, nearly 40% of genes differentially expressed between diabetic rats correspond to ESTs and predicted genes, which lack unambiguous annotation but represent attractive DN candidates, in particular when they map to rat chromosomal regions conserved with DN susceptibility loci.

### Replication of gene expression changes using Illumina BeadChips

To evaluate the robustness of transcription changes detected with Affymetrix arrays, gene expression profiling was repeated with the same samples using a different technology (Illumina BeadChips). Technical aspects of Affymetrix and Illumina array data processing (signal extraction and normalisation) and cross platforms consistency of results in terms of fold-change of expression and statistical significance, have been previously addressed [24]. Of the 1,182 transcripts found differentially regulated in at least one of the comparisons (714 known genes and 468 ESTs), over 750 could be unambiguously identified on the Illumina array, including 80% of known genes (566 genes) and 41% of ESTs (193 ESTs) (Additional file 6). In all comparisons, replication of Affymetrix results with Illumina in terms of both statistical significance of differential expression and direction of transcriptional change was remarkably high for known genes, ranging from 83% to 90%, whereas replication rate for ESTs was more modest (56% to 70%). Lower replication rate of Affymetrix results by Illumina for ESTs than known genes may reflect ambiguous annotations of ESTs. In exceptional cases (*Dpt*, *Hmox2*, *Pde4d*, *Slc12a3*, *Stat3*, *Wdfy1 *and 7 ESTs) the directions of expression changes given by Illumina and Affymetrix were inconsistent. Large numbers of genes were found differentially expressed with Illumina but not Affymetrix despite very similar expression ratio. This can be explained by the use of technical replicates for the Illumina experiments rather than technical features of the platforms.

### Identification of DN functional and positional candidates

To study more specifically expression of genes localized in DN susceptibility loci, we initially used comparative genome mapping data [29] to determine that regions of rat chromosomes 2q24-25, 2q31-32, 3q36-43, 8q12-13, 8q31-32, 11q22-23, 13p12-13, 18p12-13 and 18q12-13 show evidence of synteny conservation with DN loci in human 3q23-q29, 7q32.3-q33, 18q22-q23 and 20p12.3-p13. Using EnsEmbl genome annotation and the EnsMart browser , we selected positional candidate genes and predicted protein coding sequences at these loci to investigate their expression regulation in the Affymetrix datasests. There are over 330 annotated genes and ESTs at these loci, of which over 210 (65%) are represented on the Affymetrix array. A total of 34 DN positional candidates were differentially expressed in at least one of the comparisons between diabetic rats and controls (Table 1)(Additional file 7). These include candidates already tested in DN genetic studies (*Cndp1, Slc2a2*) and genes known to be exclusively or abundantly expressed in kidney, such as genes encoding enzymes of the polyol pathway (*Akr1b4, Akr1b8*), a kidney specific urea transporter (*Slc14a2*), a component of renal tight junctions (*Cldn16*), a zinc metalloendopeptidase (*Mep1b*) and a marker of tubular injury (*Rbp1*). Altered transcription of other genes at the loci underlines their possible role in DN etiology and pathogenesis (Table 1).

**Table 1:** Genes differentially expressed between diabetic models and controls that map to regions conserved with human DN susceptibility loci.

			GK vs WKY		STZ vs WKY		STZ vs GK	
Gene Symbol	Gene description	Genbank	TR	p-value	TR	p-value	TR	p-value
**Human 3q23-q29**								
*Bdh*	3-hydroxybutyrate dehydrogenase	NM_053995	+1.3	Ns	-1.2	Ns	-1.6	0.007
*Centb2*	Centaurin beta 2	BM389190	-1.4	Ns	1.0	Ns	+1.3	0.047
*Clcn2*	Chloride channel 2	NM_017137	+1.2	Ns	-1.1	Ns	-1.4	0.038
*Cldn16*	Claudin 16	NM_131905	-1.1	Ns	-1.6	0.002	-1.4	0.01
*Dgkg*	Diacylglycerol kinase gamma	NM_013126	+1.2	Ns	+1.6	0.025	+1.3	Ns
*Dnajb11*	DnaJ (Hsp40) subfamily B11	BI295873	1.0	Ns	-1.3	0.043	-1.3	Ns
*Eif4g1*	Eukaryotic translation initiation factor	BC098868	+1.1	Ns	-1.2	Ns	-1.4	0.011
*Fad104*	FAD104 (predicted)	AI176320	+1.3	Ns	-1.3	0.05	-1.7	5 × 10^-4^
*Fam43a*	Family with sequence similarity 43A	BE108405	-1.1	Ns	-1.7	0.017	-1.5	Ns
*Hrg*	Histidine-rich glycoprotein	NM_133428	-1.2	Ns	+2.0	0.034	+2.5	0.007
*Mfn1*	Mitofusin 1	AI169627	1.0	Ns	+1.7	0.046	+1.7	0.035
		AA943135	1.0	Ns	+1.5	0.018	+1.5	0.009
*Ncbp2*	Nuclear cap binding protein subunit 2	BI282103	+1.3	Ns	-1.5	0.046	-2	0.002
*Pld1*	Phospholipase D1	U69550	-1.3	Ns	-1.8	0.036	-1.4	Ns
*Pigx*	Subunit of GPI-mannosyltransferase	AI172192	+1.3	Ns	1.0	Ns	-1.3	0.043
*Rbp1*	Retinol binding protein 1	NM_012733	1.0	Ns	+2.3	3 × 10^-4^	+2.2	3 × 10^-4^
*Siat1*	Sialyltransferase 1	M83143	1.0	Ns	-1.6	0.002	-1.6	0.001
*Slc2a2*	Solute carrier family 2 member 2 (Glut2)	NM_012879	+3.0	5 × 10^-4^	+3.6	1 × 10^-4^	+1.2	Ns
*Ssr3*	Signal sequence receptor gamma	NM_031120	-1.2	Ns	+1.3	Ns	+1.6	0.003
*Zbed4*	Zinc finger, BED domain containing 4	AI170289	-1.1	Ns	-1.3	0.046	-1.2	Ns
---	Similar to protein NP_079596	AI412011	+1.4	Ns	-1.5	0.044	-2.1	0.001
**Human 7q32.3-q33**								
*Akr1b4*	Aldo-keto reductase family 1B4	NM_012498	1.0	Ns	+2.2	Ns	+2.1	0.024
*Akr1b8*	Aldo-keto reductase family 1B8	NM_173136	-6.2	6 × 10^-5^	+1.4	Ns	+8.5	8 × 10^-6^
*Slc35b4*	Solute carrier family 35 member B4	XM_216122	+1.2	Ns	-1.1	Ns	-1.4	0.010
**Human 18q12-q23**								
*Acaa2*	Acetyl-Coenzyme A acyltransferase 2	NM_130433	-1.6	0.041	-1.3	Ns	+1.2	Ns
*Brunol4*	RNA binding protein bruno-like 4	AW530502	+1.1	Ns	+1.3	0.036	+1.2	Ns
*Cndp1*	Carnosinase 1	AI231438	1.0	Ns	-3.4	3 × 10^-5^	-3.5	8 × 10^-6^
*Cndp2*	Carnosinase 2	BI279729	+1.1	Ns	-1.3	0.042	-1.4	0.01
*G0S2*	Putative lymphocyte G0/G1 switch gene	AI406939	+1.3	Ns	-1.6	Ns	-2.1	0.004
*Mapk4*	Mitogen-activated protein kinase 4	BG378232	+1.3	Ns	+1.5	0.023	+1.2	Ns
*Mep1b*	meprin 1 beta (endopeptidase-2)	NM_013183	1.0	Ns	-1.4	0.042	-1.4	0.050
*Pqlc1*	PQ loop repeat containing 1	AI228284	+1.6	0.044	+1.7	0.011	+1.1	Ns
*Slc14a2*	Solute carrier family 14, member 2	NM_019347	-1.1	Ns	+1.5	0.020	+1.7	0.005
		AF042167	-1.1	Ns	+1.4	Ns	+1.6	0.035
**Human 20p12.3-p13**								
*Cdc25b*	Cell division cycle 25 homolog B	NM_133572	+1.2	Ns	+1.6	0.028	+1.4	Ns
*Prnp*	Prion protein	NM_012631	+1.6	0.029	+1.1	Ns	-1.5	0.030

Gene expression results from Affymetrix chip hybridizations are given as transcription ratio (TR). The list of genes in these regions represented on the Affymetrix chip and not differentially expressed is given in Additional file 7. Ns, not statistically significant differences.

To provide information on renal expression of DN positional candidates not represented on the Affymetrix array, 53 genes localized in the most significant region of DN loci were selected for quantitative real time PCR (QRT-PCR) on kidney samples of diabetic and control rats. Rat orthologs of 23 of these could not be identified using EnsMart. Three genes (AGBL3, CHCHD3, LRGUK) and two ESTs (Q68DL7, Q6ZU70) are unlikely to be expressed in kidney as the oligonucleotides for the rat orthologs failed to amplify renal cDNA. Of the remaining 25 genes, 16 showed evidence of differential expression between diabetic and control rats (Table 2). With the exception of an aldo-keto reductase (*Akr1b10*), the function of these genes is largely unknown and their involvement in pathological mechanisms described in DN has not been reported.

**Table 2:** Expression regulation of rat orthologs of human genes localised in DN susceptibility loci and not represented on the Affymetrix arrays.

				**GK vs WKY**		**STZ-WKY vs WKY**		**STZ-WKY v GK**	
**Human Ensembl**	**Description**	**Symbol**	**Rat Ensembl**	**TR**	**p-value**	**TR**	**p-value**	**TR**	**p-value**
(ENSG00000)			(ENSRNO G000000)						
**Human 3q23**									
175066	Glycerol kinase 5	GK5	10942	1.00	Ns	0.66	0.026	0.67	2 × 10^-3^
114120	Solute carrier family 25, member 36	SLC25A36	39085	1.28	Ns	1.54	4 × 10^-3^	1.21	Ns
175093	SPRY domain-containing SOCS box protein 4	SPSB4	12862	0.78	Ns	0.37	5 × 10^-3^	0.47	0.013
114126	Transcription factor Dp-2 (E2F dimerization partner 2)	TFDP2	11241	0.76	Ns	1.56	Ns	2.04	Ns
114127	5'-3' exoribonuclease 1	XRN1	10027	0.71	Ns	0.66	0.030	0.93	Ns
177311	Zinc finger and BTB domain-containing protein 38	ZBTB38	12386	1.08	Ns	1.27	0.026	1.17	Ns
206250	Protein coding sequence	-	22555	0.81	Ns	0.82	Ns	1.01	Ns
**Human 7q33**									
146856	ATP/GTP binding protein-like 3	AGBL3	10397	0.95	Ns	0.52	1 × 10^-4^	0.55	3 × 10^-4^
198074	Aldo-keto reductase 1 member B10	AKR1B10	27433	1.94	0.035	0.77	Ns	0.40	1 × 10^-4^
122783	Uncharacterized protein C7orf49	C7orf49	26958	0.95	Ns	0.59	0.016	0.62	5 × 10^-3^
**Human 18q22.3-23**									
179981	Teashirt zinc finger homeobox 1	TSHZ1	16246	0.91	Ns	1.00	Ns	1.10	Ns
141665	F-box only protein 15	FBXO15	38225	0.72	Ns	0.69	Ns	0.97	Ns
130856	Zinc finger protein 236	ZNF236	16303	1.45	0.025	1.48	0.011	1.02	Ns
166540	zinc finger protein 407	ZNF407	16008	0.87	Ns	0.77	0.015	0.88	Ns
101493	Zinc finger protein 516	ZNF516	16258	1.07	Ns	1.20	Ns	1.12	Ns
**Human 20p12.3-13**									
149451	Disintegrin and metalloproteinase 33	ADAM33	21242	0.85	Ns	1.28	Ns	1.51	Ns
101222	Uncharacterized protein C20orf28	C20orf28	21247	1.19	Ns	0.80	Ns	0.67	0.016
101311	Unc-112-related protein 1 (Kindlin-1)	C20orf42	21274	0.70	0.050	0.53	2.2 × 10^-3^	0.75	Ns
125872	Uncharacterized protein C20orf75	C20orf75	32989	1.65	Ns	5.48	1 × 10^-3^	3.31	1 × 10^-3^
171984	Uncharacterized protein C20orf196	C20orf196	21268	1.40	Ns	1.22	Ns	0.87	Ns
125885	Minichromosome maintenance complex component 8	MCM8	21272	2.63	Ns	2.82	Ns	1.08	Ns
171864	Prion protein 2	PRND	21260	0.64	Ns	0.53	Ns	0.83	Ns
101236	RING finger protein 24	RNF24	21250	0.81	Ns	0.60	0.010	0.74	Ns
088827	Sialic acid-binding Ig-like lectin 1	SIGLEC1	21243	0.73	0.033	0.81	Ns	1.11	Ns
149345	Putative ubiquitin-conjugating enzyme E2 D3-like	U2D3L	13741	1.27	Ns	0.64	0.020	0.51	2 × 10^-3^

The EnsMart browser  was used to identify human-rat homologies (synteny conservation) in regions of the human genome associated with DN. Transcription ratios (TR) between models were assessed by QRT-PCR with kidney samples of GK, STZ-WKY and WKY rats previously used to hybridize microarrays. Ns, not statistically significant.

Overall, results from transcriptional analysis of DN positional candidates in the kidney of diabetic rats provided novel functional annotations (ie. transcriptional adaptations to hyperglycaemia and renal structural changes) of known and predicted genes.

### Validation of microarray data by QRT-PCR

**Figure 3:**
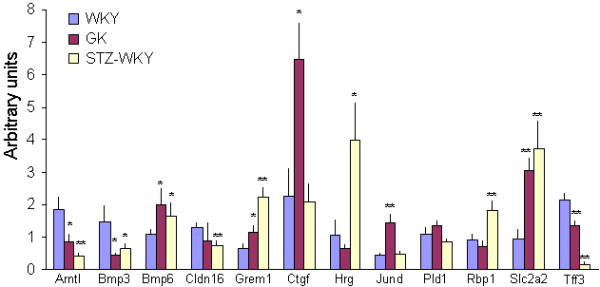
**Validation of microarray results by quantitative real time PCR**. The expression of 12 selected genes was assessed by QRT-PCR in kidney samples of GK, STZ-WKY and WKY rats previously used for microarray-based transcription profiling. Results are expressed as means ± SE, in percentage of normalized WKY values. *P < 0.05, **P < 0.01 statistically significantly different to WKY controls.

To further validate transcription changes derived by microarray platforms, QRT-PCR was carried out with 12 genes selected for their functional relevance to DN (*Bmp3*, *Bmp6*, *Grem1*, *Ctgf*, *Slc2a2*) or Type 2 DM (*Arntl*, *Slc2a2*), their location in DN susceptibility loci (*Cldn16*, *Hrg*, *Pld1*, *Rbp1*, *Slc2a2*) or the magnitude of the transcriptional change (*JunD*, *Slc2a2, Tff3*) (Figure 3). Results generally confirm microarray data including the direction of expression changes. Downregulation of *Bmp3 *expression in GK and upregulation of *Grem1 *in STZ-WKY confirm Illumina results.

## Discussion

We report renal genome-wide gene expression changes induced by prolonged hyperglycaemia in models of experimentally-induced (STZ-WKY) or spontaneous (GK) diabetes, widely used in diabetes research and known to develop renal alterations [18,20,30]. Two well-established transcription profiling technologies (Affymetrix and Illumina) provided highly concordant information on the effects of severe (STZ-WKY) or moderate (GK) hyperglycaemia, as well as GK-specific diabetes susceptibility genes, on the expression of over 15,000 known and predicted genes. Differentially expressed genes, which reflect mechanisms responsive to hyperglycaemia, provide improvements in functional annotations of known and predicted genes and potentially novel targets in DN genetics.

Renal histological changes in GK and STZ-WKY rats are relatively mild in these models [21,30-32], which may therefore be useful for investigating early molecular adaptations to hyperglycaemia in DN progression. Genome-wide gene expression profiling, which allows unbiased analysis of thousands of genes regardless of their function and chromosomal position, is a practical approach to comprehensively assess perturbed mechanisms in GK and STZ-WKY rats maintained in strictly identical conditions and uncover renal molecular adaptations to impaired glucose homeostasis in a context where environmental influences and genetic polymorphisms are minimized, as inbred WKY and GK strains share extensive genetic similarities outside GK-specific diabetes variants [19,32]. Gene expression results from two well-established and robust microarray technologies designed to investigate the transcription of largely overlapping series of genes [24], which provide systems for assessing data replication, showed remarkable concordance in transcription regulation patterns. Some discordant results may be explained by the design of the probesets (Affymetrix) or the oligonucleotides (Illumina) in different gene isoforms or the existence of polymorphisms in the sequences arrayed on the chips [33].

Transcriptomic alterations were more profound in STZ-WKY than in GK and involved largely non-overlapping sets of genes, thus underlining the importance of investigating gene expression in very different, but complementary, contexts of diabetes aetiology and pathogenesis. Age may have a confounding impact on these processes as, for obvious reasons, the models used were not age-matched, but the duration of spontaneous (GK) or experimentally-induced (STZ-WKY) hyperglycaemia was identical (3 months). Along the same line, gene expression differences between STZ-WKY and WKY, which compare pre- and post-drug treated groups and test molecular responses to prolonged and severe hyperglycaemia, may also reflect to some extent "normal" biological adaptations to ageing described in WKY [34]. Similar model-specific patterns of gene expression regulation was observed in renal transcriptomes of mouse models of STZ-induced and spontaneous (*db/db*) diabetes [31]. Diabetes-predisposing variants in the GK and *db/db *strains may therefore affect renal changes through specific mechanisms not necessarily related to hyperglycaemia itself. The existence of such specific polymorphisms [35] and a genetic predisposition to salt-induced hypertension [36] are documented in the GK strain. Metabolic and hormonal factors, as well as renal structural alterations secondary to impaired glucose regulation, may also explain the extent of renal transcriptomic differences between STZ-WKY and GK. Insulin deficiency is the leading cause of hyperglycaemia in STZ-treated animals [37], whereas the GK strain develops insulin resistance, which is associated with DN [38] and may play a specific role on renal gene expression in GK rats.

Combining transcriptomic results in GK and STZ-WKY rats with published data in animal models of diabetes [31,39,40]. and in humans [41,42] contribute to the enrichment of renal functional pathways that may be involved in DN. Of note, upregulation of *Grem1*, a bone morphogenetic protein antagonist, and strong downregulation of cadherins, *Egf *and *Tff3*, a protein promoting epithelial cell restitution, may contribute to renal damage in STZ-WKY, [43,44]. In GK rats, altered transcription of proliferation and differentiation factors (*Bmp3*, *Bmp6*, *Bmp7*) [45] suggests the involvement of mechanisms protecting the kidney against interstitial fibrosis [20], that upregulation of transcripts increasing extracellular matrix production (*Ctgf*, *Mmp9*) may initiate [46]. Downregulation of aldo-keto reductases (*Akr1b8, Akr1c12, Akr7a3*) and upregulation of *Sord *(in STZ-WKY) may reflect the activation of compensatory mechanisms preventing the accumulation of sorbitol and reducing polyol pathway flux [26]. Stimulated expression of glutathione synthase in GK may reflect cytoprotective mechanisms, whereas its downregulation in STZ-WKY may be balanced by increased expression of glutathione transferases, therefore contributing to protect the cells against oxidative stress [47]. However, expression of genes encoding protein known to play a role in DN pathogenesis, including TGFB, heme oxygenase (HMOX1), osteopontin (SPP1), were not significantly altered in diabetic rats, despite a 30–40% upregulation.

The primary aim of the study was to identify genes that are differentially expressed in diabetic models and map to regions of the rat genome that show evidence of synteny conservation with human loci associated with DN. As largely overlapping series of genes are represented on Illumina and Affymetrix arrays, replicated gene transcription differences with the two technologies provide robust information for selecting positional and functional DN candidate genes. Candidates include genes and ESTs that have no apparent functional relevance to DN, but their renal transcription patterns indicate a possible role in kidney structural and metabolic alterations. Of note, hepatic transcription profiling data in the same animals show that only a small proportion (6%–11%) of genes are consistently differentially expressed in kidney and liver (data not shown). Comparative genomic analysis of DN positional candidates mapped to 3q, 7q, 18q and 20p [8-11] revealed several differentially expressed genes that have not been tested in genetic studies of DN [13,27,28] and may therefore be important in genetic and clinical investigations of DN. Among relevant genes, *Slc14a2 *encodes a vasopressin-dependent urea transporter expressed in the collecting duct. Its upregulation in STZ-WKY may contribute to nitrogen conservation in response to glucosurea [48]. In these rats, downregulation of *Cldn16*, a major structural component of tight junction in the ascending limb of Henle, may result in ions wasting and renal glomerular and tubular alterations [49,50]. Finally, altered renal transcription of *Rbp1 *and *Mep1b *has been reported in mouse models of experimentally-induced diabetes [31] and tubular fibrosis [51].

Positional candidates differentially expressed in diabetic rats also include protein coding sequences predicted by bioinformatic models which nevertheless represent important functional DN candidates. Even though genome annotations and microarray technologies keep improving, potentially important DN positional candidate genes are not represented on gene expression arrays. Systematic renal expression analysis of ESTs mapped to the most significant regions of DN loci identified transcripts differentially regulated between GK and STZ-WKY rats. Of note, using quantitative RT-PCR, we found evidence of significant changes in expression for transcripts predicted to encode a further aldo-keto reductase (*Akr1b10*) and the zinc finger protein 236, which has been tested as a DN candidate [52]. In the vast majority of cases, confirmation of gene organization and biological functions, including altered protein abundance, is required. Although we prioritized our study to gene expression studies in total kidney to take into account interactions between renal cell types in the regulation of organ function, further investigations in tubular and mesangial cells will establish tissue specific gene expression patterns.

## Conclusion

Our study illustrates a pertinent approach utilizing both functional and positional criteria for selecting disease candidate genes. It provides a comprehensive survey of differential renal transcription adaptations to prolonged severe or moderate hyperglycaemia which likely involve pathophysiological and compensatory mechanisms. Comparative genomic analysis of genome-wide gene expression data can be used to initiate human genetic studies on specific genes in that may provide entry points in etiologically important gene pathways in DN.

## Abbreviations

DM: Diabetes Mellitus; DN: Diabetic Nephropathy; EST: expressed sequence tag; GK: Goto-Kakizaki; IPA: Ingenuity Pathway Analysis; LIMMA: Linear Models for Microarray Analysis; RMA: Robust Multi-array Analysis; STZ: Streptozotocin; WKY: Wistar-Kyoto; QRT-PCR: quantitative real time PCR; TR: transcription ratio.

## Competing interests

The authors declare that they have no competing interests.

## Authors' contributions

YH, PYW and MTB performed comparative genomic analysis and qRT-PCR. CB and MTB performed Affymetrix-based gene transcription profiling. PJK and KA performed Illumina-based gene transcription studies. KA and KJW carried out phenotypic and histological analyses of the rat models. SPW and MTB carried out statistical analyses, bioinformatic studies, gene annotations of microarray data and biological data interpretation. MTB, NV and DG drafted the manuscript. LT, PHG, SH, MM, HHP, MF, RDC, ML, NV, MTB and DG conceived of the study, and participated in its design and coordination. All authors read and approved the final manuscript.

## Pre-publication history

The pre-publication history for this paper can be accessed here:



## Supplementary Material

Additional file 1 **Oligonucleotides designed for QRT-PCR analysis of renal gene transcription regulation in diabetic and control rats**. Details of symbols, descriptions and GenBank accession numbers of genes tested for differential expression between GK, STZ-WKY and WKY rats using QRT-PCR, alongwith primer sequence and PCR product length. Click here for file

Additional file 2 **Phenotypic features of GK, STZ-WKY and WKY rats**. Body weight and plasma glucose concentrations in GK, STZ-WKY and WKY rats. Click here for file

Additional file 3 **Photomicrographs of PAS-stained kidney sections from GK, STZ-WKY and WKY rats (×400)**. Renal histopathological features in the diabetic strains (GK, STZ-WKY) and in WKY normoglycaemic controls. Click here for file

Additional file 4 **Details of genes differentially expressed in kidneys of GK and STZ-WKY models of diabetes and WKY controls**. Details of Affymetrix probeset references, symbols and descriptions of genes found significantly differentially expressed (P < 0.05) between diabetic rats (GK, STZ-WKY) and WKY controls, alongwith calculation of transcription fold change and statistical significance of transcriptional changes. Click here for file

Additional file 5 **Overview of renal transcriptomic changes in pathways underlying differential gene expression adaptations to moderate or severe hyperglycaemia in rat models of diabetes**. Selection of the most significant functionally related groups of genes found differentially expressed between diabetic rats (GK, STZ-WKY) and WKY controls. Click here for file

Additional file 6 **Comparison of Affymetrix and Illumina kidney gene expression profiling data for genes found differentially expressed between GK, STZ-WKY and WKY rats using the Affymetrix platform**. Replication analysis of Affymetrix-based gene expression changes between GK, STZ-WKY and WKY rats using data from Illumina arrays showing fold-changes and statistical significance of differential expression derived by the two microarray technologies. Click here for file

Additional file 7 **Genes mapped to chromosomal regions of the rat genome conserved with human DN loci and found not differentially expressed between diabetic models**. Details of Affymetrix probeset references, symbols and descriptions of rat genes localised in genomic regions homologous to human DN susceptibility loci, and not significantly differentially expressed (P > 0.05) between diabetic rats (GK, STZ-WKY) and WKY controls, alongwith calculation of transcription fold change and statistical significance of transcriptional changes. Click here for file
