# Supplementary material for: Functional annotations of diabetes nephropathy susceptibility loci through analysis of genome-wide renal gene expression in rat models of diabetes mellitus
Source: BMC Med Genomics. 2009 Jul 9;2:41. doi: 10.1186/1755-8794-2-41 (PMC2717999; doi:10.1186/1755-8794-2-41)
Supplement: Additional file 2 — Phenotypic features of GK, STZ-WKY and WKY rats. Body weight and plasma glucose concentrations in GK, STZ-WKY and WKY rats. [file 1755-8794-2-41-S2.doc]

**Additional file 1.** Phenotypic features of GK, STZ-WKY and WKY rats.

|  | WKY | GK | STZ-WKY |
| --- | --- | --- | --- |
| Plasma glucose (mM) | 7.4 ± 0.6 (6) | 11.8 ± 1.0* (8) | 15.2 ± 4.6* (6) |
| Body weight (g) | 316 ± 47 (6) | 291 ± 26 (8) | 262 ± 31* (6) |

Phenotypes were recorded in 3 months old WKY and GK rats, and 6 months old STZ-WKY rats. Number of rats is reported in parentheses. Data are means ± SE. *P<0.05 significantly different to WKY rats.
