# Supplementary figures and images for: Functional annotations of diabetes nephropathy susceptibility loci through analysis of genome-wide renal gene expression in rat models of diabetes mellitus
Source: BMC Med Genomics. 2009 Jul 9;2:41. doi: 10.1186/1755-8794-2-41 (PMC2717999; doi:10.1186/1755-8794-2-41)

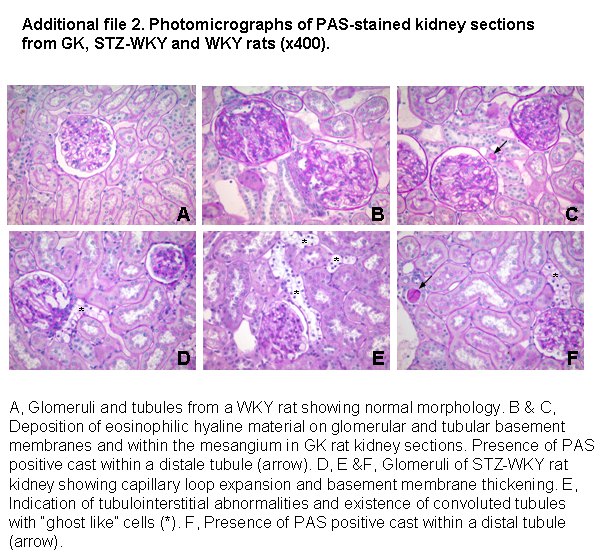

Supplement: Additional file 3 — Photomicrographs of PAS-stained kidney sections from GK, STZ-WKY and WKY rats (×400). Renal histopathological features in the diabetic strains (GK, STZ-WKY) and in WKY normoglycaemic controls. [file 1755-8794-2-41-S3.tiff]
