# Supplementary material for: Functional annotations of diabetes nephropathy susceptibility loci through analysis of genome-wide renal gene expression in rat models of diabetes mellitus
Source: BMC Med Genomics. 2009 Jul 9;2:41. doi: 10.1186/1755-8794-2-41 (PMC2717999; doi:10.1186/1755-8794-2-41)
Supplement: Additional file 5 — Overview of renal transcriptomic changes in pathways underlying differential gene expression adaptations to moderate or severe hyperglycaemia in rat models of diabetes. Selection of the most significant functionally related groups of genes found differentially expressed between diabetic rats (GK, STZ-WKY) and WKY controls. [file 1755-8794-2-41-S5.doc]

**Additional file 3: Overview of renal transcriptomic changes in pathways underlying differential gene expression adaptations to moderate or severe hyperglycaemia in rat models of diabetes.**

|  | **Comparisons** | |
| --- | --- | --- |
| Annotation | GK vs WKY | STZ-WKY vs WKY |
|  |  |  |
| Molecular and cellular | Cell to cell signaling and interactions (34) | Amino acid metabolism (28) |
| functions | Protein degradation (10) | **Small molecule biochemistry (76)** |
|  | Cellular growth and proliferation (32) | **Carbohydrate metabolism (21)** |
|  | **Carbohydrate metabolism (11)** | Cell morphology (35) |
|  | **Small molecule biochemistry (29)** | Molecular transport (45) |
|  |  |  |
| Physiological system | Immune response (27) | Organ development (16) |
| development | Immune and lymphatic system development and function (27) | Reproductive system development and function (14) |
| and function | **Tissue morphology (12)** | **Organismal development (32)** |
|  | Skeletal and muscular system development and function (19) | **Tissue morphology (24)** |
|  | **Organ development (25)** | Hair and skin development and function (12) |
|  |  |  |
| Canonical pathways | Antigen presentation pathway | Glutathione metabolism |
|  | Pyruvate metabolism | **Glycine, serine and threonine metabolism** |
|  | **Glycine, serine and threonine metabolism** | Histidine metabolism |
|  | Starch and sucrose metabolism | Arginine and proline metabolism |
|  | Phenylalanine metabolism | Fatty acid metabolism |

Ingenuity Pathways Analysis (Ingenuity Systems, Mountain View, CA, USA) knowledgebase was used for biological interpretation of Affymetrix transcriptome data in GK, STZ-WKY and WKY rats. Pathways and mechanisms affected in both diabetic models are reported in bold. Pathway analysis illustrates gene expression mechanisms consistently affected in both diabetic models (eg. carbohydrate metabolism, tissue morphology and development) and others that are predominantly altered in the GK (cellular signalling and immune response) or STZ-WKY rats (metabolism of several amino acids). Numbers of genes differentially expressed are in parentheses.
